# ΔNp73 enhances HIF-1α protein stability through repression of the ECV complex

**DOI:** 10.1038/s41388-018-0195-2

**Published:** 2018-04-09

**Authors:** Marina Stantic, Johanna Wolfsberger, Habib A. M. Sakil, Margareta T. Wilhelm

**Affiliations:** Department of Microbiology, Tumor and Cell Biology (MTC), Karolinska Institutet, 171 77 Stockholm, Sweden

## Abstract

Cellular responses to low oxygen conditions are mainly regulated by the Hypoxia-inducible factors (HIFs). Induction of HIF-1α in tumor cells activates the angiogenic switch and allows for metabolic adaptations. HIF-1α protein levels are tightly regulated through ubiquitin-mediated proteosomal degradation; however, high levels of HIF-1α is a common feature in many solid tumors and is thought to enhance cancer cell proliferation, migration, and survival. Here, we report that the oncogenic p73 isoform, ∆Np73, increases HIF-1α protein stability. We found that ∆Np73 represses expression of genes encoding subunits of the ECV complex, in particular Elongin C, Elongin B, Cullin 2, and Rbx1. The ECV complex is an E3 ligase complex responsible for polyubiquitinating HIF-1α. Loss of ∆Np73 increases ubiquitination of HIF-1α, leading to its degradation via the proteosomal pathway, and subsequent decrease of HIF-1α target genes. Taken together, our data demonstrates that high levels of ∆Np73 stabilize HIF-1α protein, allowing for it to accumulate and further potentiating its transcriptional activity and supporting tumor progression.

## Introduction

The hypoxia-inducible factor (HIF) family of transcription factors are the main mediators of the transcriptional response to oxygen deprivation [1]. The HIF family consists of basic helix-loop-helix-PER-ARNT-SIM (bHLH-PAS) proteins that form a heterodimeric complex consisting of an α-subunit (HIF-1α, HIF-2α, and HIF-3α) that is rapidly degraded in the presence of oxygen, and a stable beta-subunit (HIF-1β). Upon decreasing oxygen levels the α-subunit is stabilized, binds to HIF-1β, and translocates into the nucleus where the protein complex binds to hypoxia-response elements with the consensus sequence G/ACGTG in target genes and activates transcription. HIF target genes are functionally involved in proliferation, survival, erythropoiesis, glucose metabolism, mitochondrial integrity, angiogenesis, invasion, and metastasis [2]. HIF-1α levels are often high in human tumors and its expression has been correlated with poor patient outcome in a wide variety of tumors including breast, pancreatic, cervical, rectal, ovarian, and bladder cancer [3–9]. Intratumoral hypoxia and elevated levels of HIF-1α have been correlated with poor prognosis in breast cancer patients, and is linked to an increase in tumor size, lymph node metastasis, tumor stage, and histological grade. Furthermore, elevated HIF-1α levels are also associated with HER2 overexpression, as well as increased VEGF, COX-2, and Ki67 levels, suggesting that HIF-1α is strongly linked to more aggressive forms of breast cancer [10].

A dynamic interaction between HIF-1α and the ubiquitination machinery determines its cellular protein levels. Within well-oxygenated tissues, or normoxia, HIF-1α is hydroxylated by the prolyl-4-hydroxylase domain (PHD) enzymes PHD1 (EGLN2), PHD2 (EGLN1), or PHD3 (EGLN3) in an oxygen-dependent manner. The PHDs hydroxylate specific proline residues (Pro402/Pro564) within the oxygen-dependent degradation domain of HIF-1α. The hydroxylated proline residues are recognized by the von Hippel–Lindau protein (pVHL), thus facilitating the interaction of HIF-1α with pVHL. pVHL is the substrate recognition subunit of an E3 ubiquitin ligase complex that also include Cullin 2, Elongin B, Elongin C, and Rbx1 and is collectively called the ECV complex [11]. The ECV complex catalyzes the poly-ubiquitination of HIF-1α which targets it for degradation via the 26S-proteasome [12]. In hypoxic conditions, in contrast, HIF-1α’s half-life is greatly increased. The reduction of molecular oxygen concentration inhibits the activity of the PHD enzymes and HIF-1α is no longer prolyl hydroxylated and thus not recognized by pVHL, which leads to HIF-1α accumulation, heterodimerization to HIF-1β, and translocation into the nucleus.

The TP73 gene belongs to the p53-family that also includes TP53 and TP63. The TP73 gene encodes for full-length proteins, TAp73α−η, that share structural and functional homology with p53 and act as tumor suppressors. The usage of an intrinsic promoter results in NH2-terminally truncated dominant-negative isoforms, ∆Np73α−η, which have been shown to act as oncogenes. High expression levels of ∆Np73 have been implicated in a number of solid cancers, such as medulloblastoma, ovarian, lung, colon, and breast cancer [13–16], and correlate with poor prognosis and chemo-resistance in patients [17–19].

Recently, we and others have shown that HIF-1α protein stability and transcriptional activity is inhibited by TAp73 [20, 21]. Upon TAp73 loss, HIF-1α is stabilized in normoxic conditions, which is further enhanced during hypoxia. This leads to an upregulation of pro-angiogenic HIF-1α target genes, an increase in tumor angiogenesis, and enhanced tumor development [20, 21]. In addition, we also demonstrated that ∆Np73 enhances tumor angiogenesis, a finding later reproduced by two other independent studies [21–23]. Interestingly, we observed that ∆Np73 loss leads to reduced HIF-1α protein levels in E1A/Ras-transformed mouse embryonic fibroblasts (MEF) [21]; however, the mechanism as to how ∆Np73 regulates HIF-1α protein stability is not understood.

Here, we report that loss or reduction of ∆Np73 destabilizes HIF-1α protein by enhancing its ubiquitination and proteosomal degradation. Furthermore, we demonstrate that ∆Np73 represses expression of the ECV subunit proteins Elongin B, Elongin C, Cullin 2, and Rbx1, thus impairing the ubiquitination of HIF-1α, leading to its stabilization and accumulation.

## Results

### ΔNp73 knockdown (KD) reduces HIF-1α protein levels in breast cancer cells

**Fig. 1:**
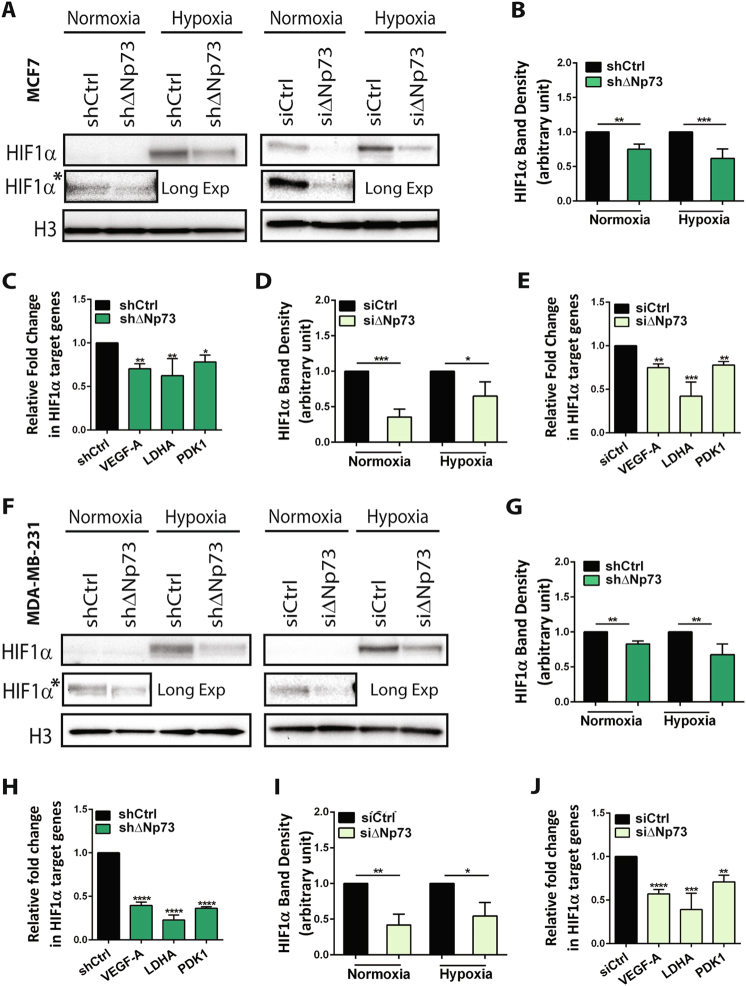
Loss of ΔNp73 reduces HIF-1α protein levels in breast cancer cell lines. Western blot analysis showing reduced protein levels of HIF-1α in ΔNp73 KD breast cancer cell lines; **a** MCF7 and **f** MDA-MB- 231 (left panel; shΔNp73, right panel; siΔNp73) cells and their respective controls (shCtrl and siCtrl). Panel HIF-1α* depicts HIF-1α bands detected in normoxic conditions after longer exposure of the membrane. **b**–**d**, **g**–**i** HIF-1α band densities in normoxia and hypoxia were quantified (ImageLab software) and normalize to loading control bands (H3), then plotted as a ratio (arbitrary unit) compared to controls (shCtrl and siCtrl). Results are shown as mean ± SD (*n* = 3/group). **c**, **e**, **h**, **j** HIF-1α target genes downregulated in ΔNp73 KD MCF7 and MDA-MB-231 cells compared to controls (shCtrl and siCtrl). Relative expression was calculated using the ΔΔCT method; results shown are the mean fold change ± SEM relative to control

We have previously shown that HIF-1α protein levels, but not mRNA levels, are reduced in ∆Np73^−/−^ MEFs [21]. To investigate this further, we knocked down ∆Np73 mRNA levels in MCF7 and MDA-MB-231 breast adenocarcinoma cells either stably, using an integrated small hairpin RNA (shRNA) targeting ∆Np73 (sh∆Np73 cells), or transiently using siRNA targeting a different region of ∆Np73 (si∆Np73 cells). Both approaches resulted in significant downregulation of ∆Np73 mRNA (Supplementary Fig. 1A–D). Next, we assessed HIF-1α protein levels after 6 h in hypoxia, and could observe reduced levels of HIF-1α protein in both MCF7 and MDA-MB-231 shΔNp73 KD cells compared to shControl (Fig. 1a, b, f, g). This was also observed in si∆Np73 cells compared to siControl (Fig. 1a, d, f, i). Interestingly, we could also observe reduced HIF-1α protein levels in ∆Np73 KD cells in normoxia (Fig. 1a, b, d, f, g, i; HIF-1α*), suggesting that ∆Np73 loss impairs HIF-1α accumulation in both normoxic and hypoxic conditions. This was accompanied by reduced expression of known HIF-1α target genes, VEGF-A, PDK1, and LDHA (Fig. 1c, e, h, j). In contrast, overexpression of ∆Np73α increased HIF-1α protein levels in normoxia and hypoxia in both MCF7 and MDA-MB-231 cells (Supplementary Fig. 2A–D), further supporting our finding that ∆Np73 is important for HIF-1α accumulation.

### Reduced HIF-1α levels in ΔNp73-deficient tumors

**Fig. 2:**
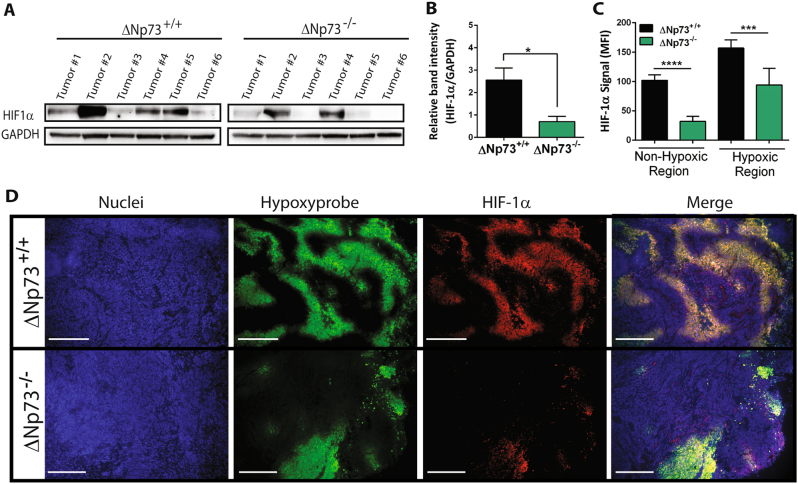
Reduced HIF-1α levels in ΔNp73-deficient tumors: **a**–**b** Representative western blot and quantification showing HIF-1α protein levels in wild-type (∆Np73^+/+^; *n* = 6) and KO ∆Np73 (∆Np73^−/−^; *n* = 6) MEF-derived tumors. **c**–**d** Mean fluorescent intensity (MFI) was determined using pixel counting for total HIF-1α signal in non-hypoxic regions (red) and the hypoxic regions where it co-localized with the HypoxyProbe-1 (green); (*n* = 5/group, five fields/tumor was used for quantification; **P < 0.01). Representative immunofluorescent images of HIF-1α (red) and hypoxia regions (green) in ΔNp73^+/+^ and ΔNp73^−/−^ tumors (scale bar, 100 μm)

To investigate the effect of ∆Np73 on HIF-1α levels in vivo, in an established tumor, we injected E1A/Ras-transformed wild-type and ΔNp73 knockout MEFs in Nude mice and followed tumor growth. As previously reported [21, 24], loss of ∆Np73 significantly impaired tumor growth (Supplementary Fig. 3A, B). Next we examined HIF-1α protein levels in the isolated tumors by western blotting and could observe reduced levels of HIF-1α protein in ∆Np73^−/−^ tumors compared to wild-type (Fig. 2a, b), which remained significant after normalizing to tumor size (Supplementary Fig. 3C), suggesting that the reduction in HIF-1α protein levels is due to loss of ∆Np73 and not due to differences in tumor size. Additionally, we analyzed HIF-1α levels in non-hypoxic and hypoxic regions of the tumors using immunofluorescence. To visualize regions of low oxygen in the tumors the mice were injected prior to sacrifice with HypoxyProbe-1, a pimonidazole HCl adduct-forming hypoxia marker that enables visualization of tissues experiencing low oxygen. We observed significantly reduced HIF-1α levels both in hypoxic and non-hypoxic regions in ∆Np73^−/−^ tumors compared to wild-type (Fig. 2c, d), demonstrating that loss of ∆Np73 reduces both hypoxic and non-hypoxic accumulation of HIF-1α in vivo.

### Loss of ∆Np73 enhances HIF-1α ubiquitination and pVHL-dependent degradation

Although HIF-1α is mainly regulated on post-translational level through the proteosomal degradation pathway, there are reports of both transcriptional and translational regulation [25]. We could not see any effect on HIF-1α mRNA levels either upon ∆Np73 KD or overexpression (Supplementary Fig. 4A–D), suggesting that ∆Np73 does not affect HIF-1α transcription but rather protein translation or stability.

**Fig. 3:**
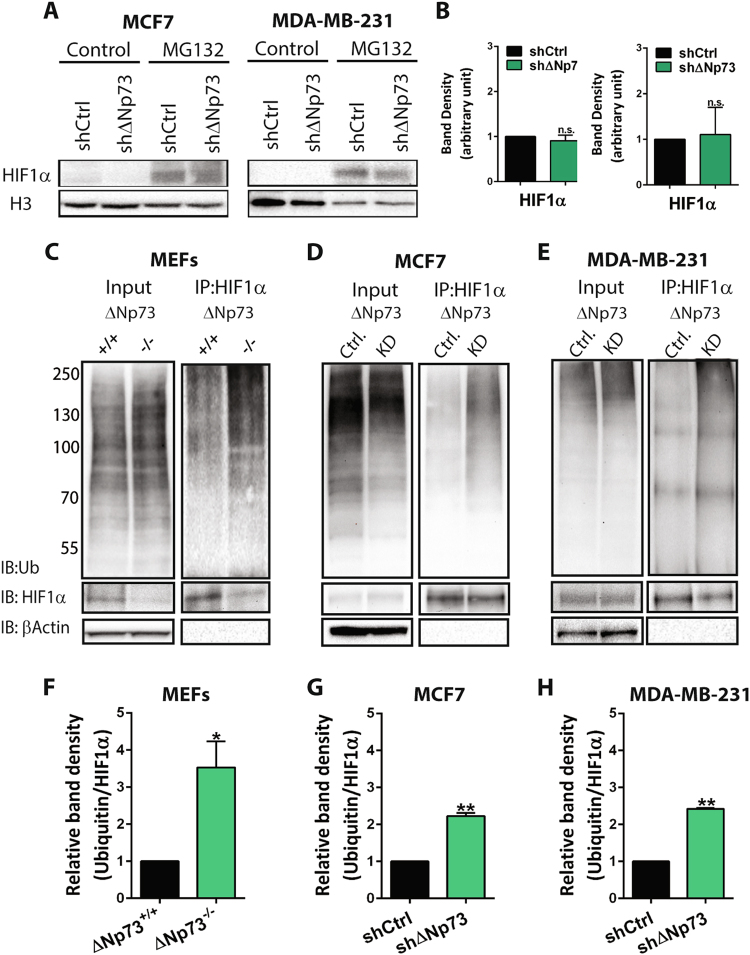
Loss of ∆Np73 enhances HIF-1α ubiquitination. **a**–**b** Representative western blot showing HIF-1α protein levels in MCF7 and MDA-MB-231 shΔNp73 and shCtrl cells after 6 h treatment with the proteosomal inhibitor MG132 (5 μM), and quantification showing HIF-1α protein levels in sh∆Np73 KD cell lines and representative controls (shCtrl). Band density for HIF-1α was quantified (ImageLab software) and normalized to their respective loading control bands (H3), and plotted as a ratio (arbitrary unit) compared to control (shCtrl). **c**–**e** MEFs^E1A/Ras^ WT (ΔNp73^+/+^) and KO (ΔNp73^−/−^), MCF7 and MDA-MB-231 shΔNp73 (KD) and shControl (Ctrl) cells were treated with MG132 for 6 h followed by immunoprecipitation (IP) with anti-HIF-1α antibody. Immunoprecipitates were analyzed by Westerns blots using anti-ubiquitin, anti-HIF-1α, and β-actin antibodies. **f**–**h** Band density for ubiquitin and HIF-1α was quantified using ImageLab software, the relative band density of ubiquitin bound to immunoprecipitated HIF-1α was determined for each cell line and shown as mean ± SD (*n* = 3/cell line)

To further study the mechanism by which ∆Np73 affects HIF-1α protein levels, we treated MCF7 and MDA-MB-231 shRNA cells with MG132, an inhibitor of the 26S proteasome-dependent degradation machinery. We observed that the effect of ∆Np73 on HIF-1α was reversed upon MG132 treatment, suggesting that ∆Np73 KD increases proteasomal degradation of HIF-1α (Fig. 3a, b). To assess HIF-1α protein stability we treated MEFs, MCF7, and MDA-MB-231 cells with MG132 and performed immunoprecipitation to assess the amount of ubiquitin bound to HIF-1α. Indeed, both in ∆Np73^−/−^ and ∆Np73 KD cells we detected an increase of ubiquitin bound to HIF-1α (Fig. 3c–h), showing that absence of ∆Np73 enhances HIF-1α ubiquitination, thereby targeting it for degradation.

**Fig. 4:**
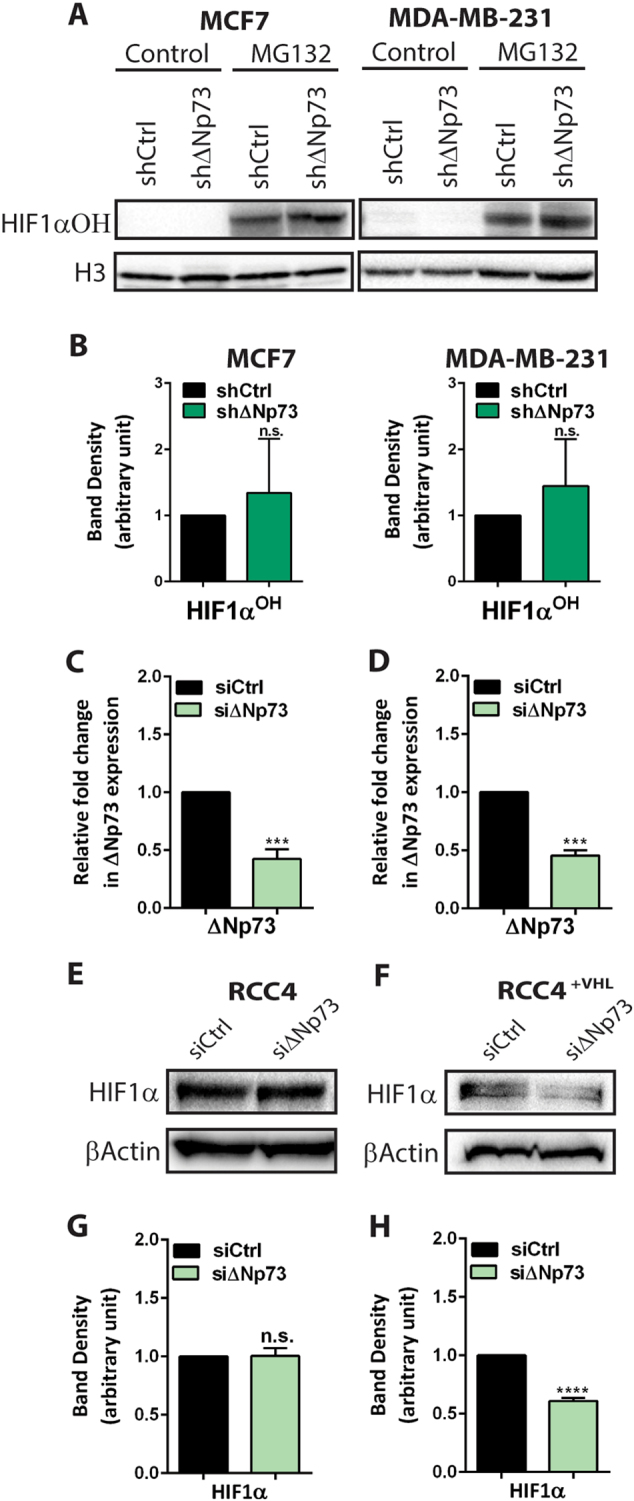
Deregulation of HIF-1α by ∆Np73 is dependent on pVHL. **a**, **b** Western blot analysis and quantification of hydroxylated HIF-1α (HIF-1α^OH^) protein levels in MCF7 and MDA-MB-231 shΔNp73 cells (green bars; shΔNp73) and corresponding controls (black bars; shCtrl) treated for 6 h with the proteosomal inhibitor MG132 (5 μM). **c**, **d** Relative fold change in ∆Np73 mRNA expression levels in RCC4 and RCC4^+VHL^ cell lines deficient for ∆Np73 (si∆Np73) compared to control (siCtrl). Relative expression was calculated using the ΔΔCT method; results shown are the mean fold change ± SEM relative to control. n.s. not significant. **e**-**h** Western blots and quantification showing HIF-1α protein levels in si∆Np73 KD RCC4 and RCC4^+VHL^ cell lines and representative controls (siCtrl). Band density for HIF-1α^OH^ and HIF-1α was quantified (ImageLab software) and normalized to their respective loading control bands (H3 or β-Actin), and plotted as a ratio (arbitrary unit) compared to control (shCtrl or siCtrl). Results are shown as mean ± SD (*n* = 3/group)

To identify by which mechanism ubiquitination increases we investigated post-translational modifications of HIF-1α. In addition to ubiquitination, hydroxylation of HIF-1α is considered the primary post-translational modification important for controlling HIF-1α protein levels. The PHD1-3 proteins catalyze the hydroxylation of proline residues within HIF-1α, enabling the interaction with pVHL and thus initiating poly-ubiquitination and proteosomal degradation of HIF-1α. Considering that PHD2 (EGLN1/SM20) has been identified as a p53 target gene [26] and it is known that ∆Np73 can inhibit p53-mediated transcription [27], we reasoned that ∆Np73 may interfere with PHD activity and HIF-1α hydroxylation. To test this, we examined the levels of hydroxylated HIF-1α (HIF-1α^OH^) in MCF7 and MDA-MB-231 shRNA cells. HIF-1α^OH^ is rapidly degraded and to be able to detect it the proteosomal degradation pathway was blocked by MG132 treatment. We could not observe any significant differences in HIF-1α^OH^ protein levels in MG132-treated sh∆Np73 KD cells compared to shCtrl cells (Fig. 4a, b), suggesting that the enhanced ubiquitination of HIF-1α in sh∆Np73 KD cells is not due to increased hydroxylation.

Next, we investigated whether the effect of ∆Np73 loss on HIF-1α is dependent on the substrate recognition subunit pVHL. Using siRNA we knocked down ∆Np73 in pVHL-mutant renal cell carcinoma RCC4 cells and pVHL-reconstituted RCC4 cells (RCC4^+VHL^) (Fig. 4c, d). We could not detect any decrease of HIF-1α levels in si∆Np73 RCC4 cells (Fig. 4e, g), while a reduction in HIF-1α levels in the si∆Np73 RCC4^+VHL^ cells compared to control was observed (Fig. 4f, h), demonstrating that the decrease in HIF-1α protein levels upon ∆Np73 KD is dependent on pVHL. However, we could not detect any changes in VHL mRNA levels in either hypoxic or normoxic conditions in MCF7 and MDA-MB-231 cells deficient for ∆Np73 compared to control (Supplementary Fig. 5A, B), indicating that although pVHL is needed for the decrease in HIF-1α levels, ∆Np73 does not affect VHL levels.

### Analysis of gene expression data identifies the ECV complex as a target for ∆Np73 in breast cancer patients

**Fig. 5:**
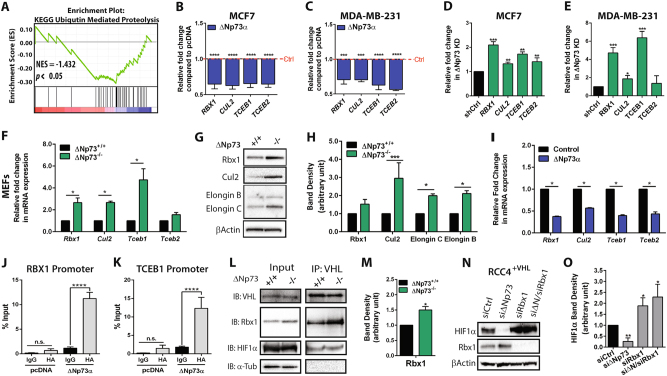
Expression analysis of ECV complex genes. **a** Enrichment plot. The vertical black lines indicate the positions of genes in the studied gene set in an ordered, non-redundant dataset. The green curve represents the enrichment score curve obtained by the GSEA software. Breast cancer samples expressing high levels of ΔNp73 show decreased expression of genes involved in Ubiquitin-mediated proteolysis, *P* < 0.05. **b**, **c** Ectopic expression of ΔNp73α in MCF7 and MDA-MB-231 cells resulted in downregulation of *RBX1*, *CUL2*, *TCEB1*, and *TCEB2*. Results shown as mean fold change ± SD relative to pcDNA (Ctrl; red dotted line, *n* = 3/group). **d**, **e** ECV gene expression analysis in ∆Np73 KD MCF7 and MDA-MB-231 (sh∆Np73) compared to control (shCtrl). **f** Gene expression analysis of ECV genes in ΔNp73^−/−^ MEFs^E1A/Ras^ compared to WT. **g**–**h** Representative Western blots and band density quantification of Rbx1, Cul2, Elongin B, Elongin C proteins in ΔNp73^−/−^ and WT MEFs^E1A/Ras^. Results are shown as mean ± SD (*n* = 3/group). **i** qRT-PCR analysis shows reduced expression of ECV complex genes, upon reconstitution of ΔNp73α in ΔNp73^−/−^ MEFs^E1A/Ras^. Samples were normalized to 28S or 18s and compared to shCtrl or WT MEFs^E1A/Ras^, respectively. Relative expression was calculated using the ΔΔCT method; results shown are the mean fold change ± SEM relative to control. **j**–**k** ChIP-qPCR for RBX1 and TCEB1 show enrichment increase (% input) in MCF7 cells with ectopic expression of pcDNA-HA-∆Np73α compared to IgG control and pcDNA (empty vector), ****P ≤ 0.0001. **l**–**m** Immunoprecipitation (IP) with anti-VHL antibody was performed on whole cell lysates from WT (ΔNp73^+/+^) and KO (ΔNp73^−/−^) MEFs^E1A/Ras^. Immunoprecipitates were analyzed by Westerns blot using anti-VHL, anti-Rbx1, anti-HIF-1α, and α-tubulin antibodies. Rbx1 band density was quantified (ImageLab software) and plotted for MEFs^E1A/Ras^ ΔNp73^−/−^ compared to WT. Band densities were normalized relative to the intensity of the VHL band intensity of the immunoprecipitation (IP:VHL; IB:VHL). **n**–**o** Western blot analysis for HIF-1α and RBX1 protein in RCC4^+VHL^, control (siCtrl), ∆Np73 KD (si∆Np73), RBX1 KD (siRbx1), and combination KD (si∆N/siRbx1) whole cell lysates. Quantification of HIF-1α band intensity (ImageLab software) compared to control (siCtrl). Band densities were normalized to their respective loading control bands (β-Actin) and expressed as a ratio (arbitrary unit). Results are shown as mean ± SD (*n* = 3/group)

Upregulation of ∆Np73 is a common event in many types of cancer, and has been correlated with poor prognosis in breast cancer [17]. Using RNA-Seq data from the Cancer Genome Atlas, we recently showed that high levels of ∆Np73 expression is associated with both angiogenesis and hypoxia signatures in breast cancer patients [21]. To get an insight into how ∆Np73 may enhance HIF-1α stability we generated a ranked gene list using our previous analysis of differentially expressed genes between breast cancer patients with high vs. low ΔNp73 levels (Dataset 1) [21], and investigated which biological pathways ∆Np73 alter using the Broad Institute Gene Set Enrichment Analysis tool [28, 29]. Interestingly, expression of genes involved in Ubiquitin-mediated proteolysis was downregulated in tumors with high ΔNp73 levels (Fig. 5a), suggesting that ∆Np73 may downregulate genes involved in this biological process.

Among the candidate target genes we found several members of the ECV complex, the E3 ubiquitin ligase complex responsible for poly-ubiquitinating HIF-1α [30]. Specifically, we found the ECV complex genes encoding Elongin B (TCEB2), Elongin C (TCEB1), Cullin 2 (CUL2), and Rbx1 (RBX1) to be significantly downregulated (Supplementary Table 1). In concordance with our previous results, high ∆Np73 levels did not correlate with any significant changes in VHL or HIF-1α mRNA levels in the breast cancer patients (Supplementary Table 1). We went on to validate the gene expression of the ECV complex genes RBX1, CUL2, TCEB1, and TCEB2 in MCF7 and MDA-MB-231 breast cancer cell lines ectopically overexpressing ∆Np73, and found their downregulation to be consistent with the breast cancer patient data (Fig. 5b, c). In contrast, KD of ∆Np73 leads to an upregulation of the ECV complex genes (Fig. 5d, e). In addition, Elongin B (*Tceb2*), Elongin C (*Tceb1*), Cullin 2 (*Cul2*), and Rbx1 (*Rbx1*) were all upregulated both on mRNA and protein level in ∆Np73^−/−^ MEFs cells compared to WT cells (Fig. 5f, h). This was reversed by re-introduction of ∆Np73α into ∆Np73^−/−^ MEFs (Fig. 4i).

It has been shown that ∆Np73 can bind to TP73/TP53 response elements in gene promoters and repress transcription [27]. To understand the mechanism of how ∆Np73 regulate the ECV genes we analyzed the promoter regions of both human and mouse CUL2, RBX1, TCEB1, and TCEB2 using Pscan [31]. Interestingly, we found putative binding sites for TP73 in the promoter region of each gene (Supplementary Tables 2 and 3). Using chromatin immunoprecipitation (ChIP) assay, followed by qPCR, we observed binding of ∆Np73α to both RBX1 and TCEB1(Elongin C) promoters (Fig. 5j, k), but could not detect any binding to the predicted sites in CUL2 and TCEB2 promoters, the previously reported TP73 binding site in the p21 promoter was used as a positive control (Supplementary Fig. 5C) [32]. Taken together, this suggested to us that ∆Np73 may interfere with ubiquitination events downstream of pVHL through down regulation of the ECV subunits via direct binding to TP73 binding sites in the RBX1 and TCEB1 promoters.

To further study the effect of ∆Np73 on the activity of the ECV complex, we performed immunoprecipitation to assess the amount of Rbx1 found bound to pVHL. Rbx1 is the RING finger protein that, together with Cullin2, mediates the formation of poly-ubiquitin conjugates on HIF-1α [33]. We could not observe any differences in pVHL protein levels or amount of HIF-1α bound to pVHL; however, we found more Rbx1 bound to pVHL in ∆Np73^−/−^ MEFs compared to WT MEFs (Fig. 5l, m), indicative of a more active ECV complex.

We next determined whether the decrease of HIF-1α stability upon ∆Np73 KD was dependent on RBX1. For this purpose, we selected the RCC4^+VHL^ cell line since it expresses high levels of endogenous RBX1 and HIF-1α protein. Again, ∆Np73 KD resulted in a significant reduction of HIF-1α protein, whereas RBX1 KD led to a 2-fold increase of HIF-1α protein compared to control (Fig. 5n, o), demonstrating the importance of RBX1 in regulating HIF-1α levels in normoxic conditions. Interestingly, we could not observe any decrease of HIF-1α protein when both ∆Np73 and RBX1 were knocked down (Fig. 5n, o), showing that the effect of ∆Np73 KD on HIF-1α protein is indeed dependent on RBX1. Taken together, our data demonstrates that ∆Np73 plays an important role in regulating HIF-1α protein levels by repressing proteosomal degradation mediated by the ECV complex.

## Discussion

Solid tumors often suffer from hypoxia due to poorly formed and permeable tumor vasculature. Increasing evidence points toward that low oxygen levels not only play an important role in tumor progression, but also in promoting chemo and radio-resistance [34]. HIF-1α is one of the most important early response proteins to hypoxic stress, and is stabilized and activated in response to low oxygen condition. In addition, HIF-1α protein can accumulate in oxygenated conditions due to loss of VHL, aberrant growth factor signaling, increased transcription of HIF-1α, or mutations in succinate dehydrogenase or fumarate hydratase genes, leading to accumulation of succinate and fumarate that act as inhibitors of PHD’s, thus creating an oxygen-independent pseudohypoxic state resulting in HIF-1α stabilization [35]. HIF-1α target genes encoding for proteins that will enhance neovascularization, increase anaerobic glycolysis, decrease mitochondrial functions, and oxidative phosphorylation to allow the tumor cells to adapt to the hostile tumor microenvironment [36].

Immunohistochemical analysis of HIF-1α protein levels in primary breast cancer biopsies has demonstrated a significant association with mortality in several clinical studies [37, 38], showing the importance of HIF-1α in breast cancer progression. This has been further confirmed using conditional knock out mice, where it was demonstrated that HIF-1α was necessary for both primary mammary tumor growth and metastasis to lymph nodes and lungs [39].

We have previously shown that TAp73 and ∆Np73 have opposing roles in regulating tumor angiogenesis. TAp73 loss results in highly vascularized tumors with permeable vessels. In contrast, loss of ∆Np73 leads to avascular tumors that grow poorly [21]. We showed that the enhanced angiogenic phenotype in TAp73^−/−^ tumors was due to stabilization of HIF-1α and subsequent expression of pro-angiogenic and pro-inflammatory cytokines. Interestingly, we found that ∆Np73 loss leads to reduced HIF-1α protein levels, suggesting that TAp73 and ∆Np73 have opposing function on HIF-1α but the mechanism remained poorly understood [21]. Although HIF-1α is mainly regulated via post-translational mechanisms several reports have demonstrated that HIF-1α also can undergo transcriptional regulation via Angiotensin II, Glucose, HGF, LPS, and TNF-α signaling, as well as post-transcriptional regulation via a growing number of miRNA targeting the HIF-1α mRNA [25]. However, we could not observe any effect of ∆Np73 on HIF-1α mRNA levels either in cell lines or in breast cancer patient material. Instead, our data show that ∆Np73 regulates HIF-1α stability through direct repression of several members of the ECV complex, the E3 ligase complex responsible for HIF-1α polyubiquitination.

The ubiquitin pathway is important for regulation of protein levels and function, and involves the sequential action of ubiquitin-activating enzymes (E1s), ubiquitin-conjugating enzymes (E2s), and ubiquitin ligases (E3s). The ECV complex, a multi-protein complex, is the E3 ubiquitin ligase responsible for HIF-1α ubiquitination. It contains Cullin 2, a large scaffold protein that through adapter proteins Elongin B and Elongin C recruits the substrate receptor pVHL to its N-terminal region, and the RING finger protein Rbx1 to its C-terminal region [40]. Rbx1 (ROC1/HRT1) and Rbx2 (SAG/ROC2) both contain a RING zinc finger domain which is required for their ubiquitin ligase activity. Overexpression experiments have shown that Rbx1 and Rbx2 are capable of binding to all members of the Cullin family, but that Rbx1 preferentially binds to Cul2/pVHL [41]. RBX1 is constitutively expressed; however, very little is known about how RBX1 is regulated at the transcriptional level, we show here that ∆Np73 binds directly to TP73 response elements in the RBX1 and TCBE1 gene promoters, suggesting that ∆Np73 is repressing their transcription. RBX1 is essential for the proteosomal degradation of HIF-1α [42], and we demonstrate that RBX1 KD significantly increases HIF-1α protein levels, and in the absence of RBX1, ∆Np73 loss does not affect HIF-1α levels, suggesting that ∆Np73 mainly inhibits RBX1-mediated degradation of HIF-1α.

Intriguingly, we observed that ∆Np73 KD results in significant reduction of HIF-1α protein levels in non-hypoxic as well as in hypoxic conditions. Compared to the degradation pathways operating during normoxic conditions, less is known about hypoxic degradation of HIF-1α. However, it has been shown that both PHD2 (EGLN1) and PHD3 (EGLN3) are direct HIF target genes and that their expression increases during hypoxia suggesting a negative feedback [43, 44]. It has been speculated that their impaired function in low oxygen could be compensated in part by increased abundance [45]. Additionally, a recent report shows that hypoxic degradation of HIF-1α is dependent on ubiquitination, although it was shown to be independent of hydroxylation, pVHL, HAF, RACK1, sumoylation, or nuclear/cytoplasmic localization [46], suggesting unidentified E3 ligase are acting in this pathway. It would be interesting to investigate whether it is dependent on any of the ECV subunits regulated by ∆Np73. In addition, we consistently observed higher levels of ∆Np73α in hypoxia compared to normoxia (Supplementary Fig. 2A, B), this is consistent with previous reports that ∆Np73 is stabilized in hypoxia due to HIF-1α-mediated repression of the E3 ligase SIAH1 [22]. It also suggests that HIF-1α and ∆Np73 are engaged in a positive feedback loop where HIF-1α-mediated stabilization of ∆Np73 would further enhance HIF-1α stability, thus amplifying the hypoxic response.

Our data demonstrate that ∆Np73 protects HIF-1α from polyubiquitination and thus shields it from proteasomal-mediated degradation. Upregulation of ∆Np73 in tumors would create a pseudohypoxic state whereupon HIF-1α is stabilized and promote oncogenic activities such as angiogenesis, increased proliferation, survival, and metastasis.

## Methods and materials

### Cell culture and treatments

E1A/H-Ras^V12^-transformed ∆Np73^+/+^ and ∆Np73^−/−^ MEF and human cancer cells (MCF7 and MDA-MB-231) were cultured as previously reported [21]. MEFs, MCF7, or MDA-MB-231 were transfected with pcDNA3.1 (control), pcDNA-HA-ΔNp73α using Lipofectamine3000 (Life Technologies) according to manufacturer’s instructions. MCF7 and MDA-MB-231 cells with stable KD of ΔNp73 were generated by lentiviral transduction of a short hairpin RNA against ΔNp73 as previously described [47]. siRNA oligos used; siCtrl: Universal negative Control (Sigma-Aldrich), siΔNp73:5′-GCGCCUACCAUGCUGUACGUC[dT][dT], siRbx1: Mission esiRNA EHU055711 (Sigma-Aldrich). siRNA oligos was introduced into cells using Lipofectamine RNAiMAX according to manufacturer’s instructions (Life Technologies). For hypoxia experiment, GasPak™ Plus Anaerobic System (BD Biosciences) was used as previously reported [21].

### In vivo tumor formation, hypoxic region, and HIF-1α detection

Hypoxic regions and HIF-1α expression within tumors was studied using s.c. injections of transformed MEFs into the flank of 4–6-week-old athymic nude Foxn1nu/nu mice as described in [21]. Tumor growth was monitored for 18 days. Hypoxic regions were detected using the Hypoxyprobe-1 Plus Kit as per manufacturer’s protocol (Hypoxyprobe, Inc). Briefly, the probe was injected intravenously (60 mg/kg body weight) 90 min before tumor-bearing mice were sacrificed. Tumors were harvested, embedded in Tissue-Tek O.C.T. compound (VWR) and cryopreserved. 8 μm cryosections were fixed for 20 min in ice-cold Methanol/Acetone (1:1), permeabilized in 0.2% TritonX/PBS, and blocked in 5% Goat serum/PBST for 30 min before stained with anti-HIF-1α (1:100; Novus). Sections were incubated with Alexa568-conjugated anti-rabbit IgG antibody (1:500, Thermo Scientific). Thereafter, sections were stained with FITC-conjugated hypoxyprobe-1 monoclonal antibody (1:500, Hypoxyprobe™) and mounted using ProLong^®^ Diamond Antifade Mountant (Molecular Probes™). Co-localization of Hypoxic regions and HIF-1a-positive regions were determined using ImageJ softwear. All animal experiments were conducted in accordance with Karolinska Institutet guidelines and approved by Stockholm’s North Ethical Committee of Animal Research (ethical permit N214/15).

### Western blot and co-immunoprecipitation

Cell and tumor lysates were prepared in RIPA buffer (Sigma-Aldrich) containing 1× or 2× protease inhibitor, respectively (Thermo Scientific) from either normoxic, 6 h-treated hypoxia, or 5 μM MG132 (Sigma-Aldrich) cell cultures or 5 mg of tumor tissue for western blot analysis. For co-immunoprecipitation cells were treated with 5 μM MG132 for 6 h, washed with ice-cold PBS, and lysed in ice-cold cell lysis buffer (50 mM Tris HCl, pH 8.0, 150 mM NaCl, 1 mM EDTA, 1% Nonidet P-40, and 10% glycerol with protease inhibitor cocktail) for 1 h, with occasional gentle agitation. All lysates were centrifuged at 12,000 rpm for 15 min at 4 °C, supernatant collected and protein concentration determined. For immunoprecipitation, 300 μg of cell extract was pre-cleared with Dynabeads^®^ Protein G beads (Thermo Fisher Scientific), and incubated overnight with 1 μg of antibody at 4 °C rotating. To immunoprecipitate the target antigen, 10 μl of the Dynabeads^®^ Protein G beads were added to the extract–antibody complex, incubated at RT for 20 min rotating before eluting the target antigen using Bolt^®^ LDS samples buffer (per manufactures instructions (Thermo Fisher Scientific). Elutes, 10% lysate input or RIPA buffer cell lysate (20 μg) were fractionated by SDS-PAGE (Bolt™ 4–12% Bis-Tris Plus Gels; Thermo Fisher Scientific), proteins were transferred to a nitrocellulose membrane and probed for proteins of interest. All antibodies used listed in Supplementary Table 4.

### Quantitative real-time PCR

Total RNA (1 μg) was used for cDNA synthesis using Superscript III or iScript (Thermo Fisher Scientific, BioRad). qRT-PCR was performed using ABI StepOnePlu (Thermo Fisher Scientific), according to manufacturer’s suggestions. Samples were run in triplicate and normalized to 18s or 28S RNA. Primer sequences are found in Supplementary Table 4. Relative expression was calculated using the ΔΔCT method using the StepOne2.2 Software (Thermo Fisher Scientific).

### ChIP assay

MCF7 cells were transfected with pcDNA3.1 and pcDNA-HA-ΔNp73α for 16 h to induce ΔNp73α expression. Cells were collected at 90% confluence then subjected to the ChIP assay protocol described previously [21] with minor modifications. In brief, chromatin was sheared by sonication and immunoprecipitated with 5 μg anti-HA antibody (TransCruz; Santa Cruz Biotechnology) or control immunoglobulin G (IgG) antibody (Santa Cruz Biotechnology). The immunocomplexes were captured with Dynabeads® Protein G (Life Technologies), the co-immunoprecipitated DNA fragments and input DNA were analyzed via qRT-PCR using primers spanning promoter regions of the ECV genes (Supplementary Tables 3 and 4). ChIP qRT-PCR for p21 promoter was performed as a positive control [32].

### Statistical analysis

All statistical analyses were performed using Student’s *t*-test or ANOVA, and *P < 0.05, **P < 0.01, ***P < 0.005, and ****P < 0.001 were considered statistically significant. All experiments were performed three times independent of each other, unless stated otherwise.

## Electronic supplementary material

Supplementary Information

Supplementary Figure 1

Supplementary Figure 2

Supplementary Figure 3

Supplementary Figure 4

Supplementary Figure 5

Dataset 1
