# Supplementary material for: ΔNp73 enhances HIF-1α protein stability through repression of the ECV complex
Source: Oncogene. 2018 Apr 9;37(27):3729–39. doi: 10.1038/s41388-018-0195-2 (PMC6033838; doi:10.1038/s41388-018-0195-2)
Supplement: Supplementary file 1 — Supplementary Information [file 41388_2018_195_MOESM1_ESM.docx]

**Supplementary Information**

**Supplementary Table 1**: Genes found down-regulated in breast cancer patients with high ΔNp73 (Adapted from Stantic et al., (2015). PNAS, http://doi.org/10.1073/pnas.1421697112)

| **Gene** | **FC(Log2)** | **P-value** |
| --- | --- | --- |
| TCEB1 | -0.68 | 6E-19 |
| RBX1 | -0.42 | 2E-12 |
| CUL2 | -0.22 | 9E-08 |
| TCEB2 | -0.57 | 1E-12 |
| HIF1A | 0 | 6E-02 |
| EGLN2/PHD1 | 0 | 1E-06 |
| EGLN1/PHD2 | 0 | 6E-01 |
| EGLN3/PHD3 | 0 | 3E-04 |
| VHL | 0 | 7E-01 |

**Supplementary Table 2:** List generated using Pscan of putative transcription factor binding sites in the promoter regions of the human and mouse ECV genes (CUL2, TCEB1, TCEB2, RBX1)

| **Matrix ID** | **Matrix Name** | **P-value** |
| --- | --- | --- |
| MA0144.2 | STAT3 | 0.00072536 |
| MA0137.2 | STAT1 | 0.00113012 |
| MA0098.2 | Ets1 | 0.00236492 |
| MA0144.1 | Stat3 | 0.00418291 |
| MA0014.2 | PAX5 | 0.00510478 |
| MA0088.2 | ZNF143 | 0.00595895 |
| MA0137.3 | STAT1 | 0.00639096 |
| MA0749.1 | ZBED1 | 0.00842189 |
| MA0777.1 | MYBL2 | 0.00919361 |
| MA0506.1 | NRF1 | 0.00946222 |
| MA0657.1 | KLF13 | 0.0105103 |
| MA0474.1 | Erg | 0.0115846 |
| MA0518.1 | Stat4 | 0.0126586 |
| MA0088.1 | znf143 | 0.0135666 |
| MA0740.1 | KLF14 | 0.0174046 |
| MA0736.1 | GLIS2 | 0.0181287 |
| MA0629.1 | Rhox11 | 0.0196055 |
| MA0475.1 | FLI1 | 0.0267684 |
| MA0685.1 | SP4 | 0.0295527 |
| MA0513.1 | SMAD2::SMAD3::SMAD4 | 0.03296 |
| MA0048.1 | NHLH1 | 0.0356614 |
| MA0502.1 | NFYB | 0.0364724 |
| MA0489.1 | JUN(var.2) | 0.0365024 |
| MA0469.2 | E2F3 | 0.0398598 |
| MA0912.1 | Hoxd3 | 0.0411555 |
| MA0162.1 | Egr1 | 0.0415964 |
| MA0864.1 | E2F2 | 0.0455828 |
| MA0024.3 | E2F1 | 0.0490234 |
| MA0037.2 | GATA3 | 0.0493725 |
| MA0747.1 | SP8 | 0.0558288 |
| MA0748.1 | YY2 | 0.0576856 |
| MA0095.2 | YY1 | 0.0607552 |
| MA0060.1 | NFYA | 0.0676463 |
| MA0887.1 | EVX1 | 0.074009 |
| MA0681.1 | Phox2b | 0.075272 |
| MA0713.1 | PHOX2A | 0.0753637 |
| MA0472.2 | EGR2 | 0.076668 |
| MA0733.1 | EGR4 | 0.0810151 |
| MA0007.1 | Ar | 0.0814768 |
| MA0599.1 | KLF5 | 0.0816178 |
| MA0519.1 | Stat5a::Stat5b | 0.0864372 |
| **MA0861.1** | **TP73** | **0.087993** |
| MA0014.1 | Pax5 | 0.0890658 |
| MA0794.1 | PROX1 | 0.0909671 |
| MA0510.2 | RFX5 | 0.0959999 |
| MA0904.1 | Hoxb5 | 0.0962194 |
| MA0103.1 | ZEB1 | 0.0962533 |
| MA0098.3 | ETS1 | 0.0968808 |
| MA0715.1 | PROP1 | 0.0983732 |
| MA0039.2 | Klf4 | 0.0991729 |
| MA0776.1 | MYBL1 | 0.0995074 |
| MA0697.1 | ZIC3 | 0.0995223 |
|  |  |  |
|  |  |  |

**Supplementary Table 3:** Location and Sequence of putative TP73 binding sites, generated from Pscan.

| **RefSeq** | **Name** | **Score** | **Position** | **Sequence** | **Strand** |
| --- | --- | --- | --- | --- | --- |
| hg38_refGene_NM_001198778 | human CUL2 | 0.714 | -378 | GGCCAGGCTGGTCTCGAA | - |
| hg38_refGene_NM_014248 | human RBX1 | 0.790 | -628 | GCCCTGCCTTTACTCGCC | - |
| hg38_refGene_NM_005648 | human TCBE1 | 0.835 | -258 | TACAAGTCCCAGGATGCA | + |
| hg38_refGene_NM_007108 | human TCBE2 | 0.824 | -27 | GGCATGCTGGGCCACGCG | + |
| mm10_refGene_NM_029402 | mouse Cul2 | 0.847 | -688 | AGCAAGCAAACACACGCC | - |
| mm10_refGene_NM_019712 | mouse Rbx1 | 0.707 | -491 | ACCAGGCCTTGGTAAGCC | + |
| mm10_refGene_NM_001310470 | mouse Tcbe1 | 0.829 | -145 | CACAAGTCCCAAGATGCA | + |
| mm10_refGene_NM_026305 | mouse Tcbe2 | 0.744 | -58 | AGCACGCTGGGTCGCGCT | + |

**Supplementary table 4**: Antibodies and primer sequences used in Western blot, qRT-PCR and ChIP-qPCR.

| ***Antibody*** | ***Clone*** | ***Dilution*** | ***Catalog #*** | ***Company*** |
| --- | --- | --- | --- | --- |
| p73 | ER-15 | 1:5000 | ab177239 | abcam |
| β-Actin -HRP | AC-15 | 1:5000 | ab49900 | abcam |
| Hydroxy-HIF-1α | Pro564 | 1:1000 | 3434P | Cell Signaling |
| HIF-1α | - | 1:1000 | NB100-479 | Novus Biologicals |
| Cullin2 | - | 1:500 | NBP1-67535 | Novus Biologicals |
| TECB1 | - | 1:500 | NB10078353 | Novus Biologicals |
| Histone 3 | - | 1:10000 | ab1791 | Novus Biologicals |
| Ubiquitin | A-5 | 1:1000 | sc-166553 | Santa Cruz Biotechnology |
| Rbx1 | E-11 | 1:200 | sc-393640 | Santa Cruz Biotechnology |
| Elongin B | FL-118 | 1:200 | sc-11447 | Santa Cruz Biotechnology |
| VHL | G-5 | 1:500 | sc-17780 | Santa Cruz Biotechnology |
| α-Tubulin | DM1A | 1:1000 | sc-32293 | Santa Cruz Biotechnology |
| HAx TransCruz | F-7 | 5µg | sc-7392 | Santa Cruz Biotechnology |
| Normal mouse IgG | Control IgG | 5ug | sc-2025 | Santa Cruz Biotechnology |

| **qRT-PCR Primers** | | |
| --- | --- | --- |
| **Gene - Human** | **Forward 5’-3’** | **Reverse 5’-3’** |
| RBX1 | TTGTGGTTGATAACTGTGCCAT | GACGCCTGGTTAGCTTGACAT |
| CUL2 | ACGACAATAAAAGCCGTGGTC | GGATAGGCCACACATAAAGCAT |
| TCEB1 | CATCAGGCACGATAAAAGCCA | GCTGTTAGTGTAGCGAACCTTG |
| TCEB2 | CGA ACTGAAGCGCATCGTC | TCCAAGAGTTGGTCATCCTTGT |
| VHL | GGAGCCTAGTCAAGCCTGAGA | CATCCGTTGATGTGCAATGCG |
| HIF1A | GAACGTCGAAAAGAAAAGTCTCG | CCTTATCAAGATGCGAACTCACA |
| ΔNp73 | GGCTGCGACGGCTGCAGGCC | CAGGCGCCGGCGACATGG |
| VEGFA | GTCTTCACTGGATGTATTTGAC | AAAAGATCATGCCAGAGTCTC |
| PDK1 | CGGGTCGTTATGAGAGTCGA | GGGACAGCAGCCTTAATCCT |
| LDHa | TATTAGGCTATTCTTGGGCAAC | CGGGTCGTTATGAGAGTCGA |
| 28S | TTGAAAATCCGGGGGAGAG | ACATTGTTCCAACATGCCAG |
| **Gene - Mouse** | **Forward 5’-3’** | **Reverse 5’-3’** |
| Rbx1 | GTCAGCTACTTCCGAAGAGTGT | TTGAGCCATCGAGAGATGCAG |
| Cul2 | CTACGTCAATGCAGAAGGATACG | CGGACAATAGCAGCTTGAAGA |
| Tecb1 | GAGAACATGCACTAACATCAGGA | ACATGCACACTTTTGATAGCACA |
| Tecb2 | AGACCACCATCTTTACGGACG | AGTTTTGCCATCATCAAGGAGC |
| Vhl | TGTGCCATCCCTCAATGTCG | GCACCGCTCTTTCAGGGTA |
| 18s | AGTTCCAGCACATTTTGCGAG | TCATCCTCCGTGAGTTCTCCA |
|  |  |  |
| **ChIP-qPCR Primers** | | |
| **Gene-Human** | **Forward 5’-3’** | **Reverse 5’-3’** |
| RBX1 | CAAAGGCGGTCCTAGGACTG | CTAAGGCCACACTGAGCAGG |
| CUL2 | GAGACAGGGTTTCACCATGTTG | GGCTCACACCTATAATCCCAGC |
| TCBE1 | CTTAACACGCAGTGCGAGAAAC | GTTTCTCTGGTGGATACCGTCG |
| TCBE2 | GATCACTTAAAATGGCGGCG | CGTGGCCCAGCATGC |
| p21 | GTGGCTCTGATTGGCTTTCT | AGCCTCTTCTATGCCAGAGC |

**Supplementary Figure legends**

**Supplementary Figure 1:** (A-D) Decreased ΔNp73 mRNA expression levels in breast cancers cell lines MCF7 and MDA-MB-231 transduced with shRNA targeting ΔNp73 (A and C) or transfected with siRNA targeting ΔNp73 (B and D), compared to their respective controls (shCtrl and siCtrl).

**Supplementary Figure 2:**(A-B) Western blot showing HIF-1α protein levels in MCF7 and MDA-MB-231 cells with ectopic expression of ΔNp73α. Panel HIF-1α* depicts HIF-1α bands detected in normoxic conditions after longer exposure of the membrane. (C-D) Band densities for HIF-1α in normoxia and hypoxia were quantified (ImageLab software) and normalized to their respective loading control bands (H3), and expressed as a ratio (arbitrary unit) compared to control (pcDNA). Results are shown as mean ± SD (n=3/group

**Supplementary Figure 3:** (A) E1A/Ras^V12^-transformed WT or ΔNp73^−/−^ MEFs were injected s.c. into nude mice (*n* = 6/group), tumor growth was measured at a 3-day interval up to 18 days postinjection. Results are shown as the mean ± SEM, *P* < 0.001. (*B*) Decreased tumor weight in absence of ΔNp73 (ΔNp73^−/−^, 0.137g ± 0.042g vs. WT, 0.586g ± 0.074g; ****P* < 0.005) Results are shown as the mean ± SD. (C) HIF-1α protein levels normalized to individual tumor volumes in ΔNp73 deficient tumors compared to WT. Results are shown as the mean ± SD (ΔNp73^−/−^, 0.20 ± 0.0792 vs. WT, 0.677 ± 0.0; ***P* < 0.005).

**Supplementary Figure 4**: Relative fold changes in HIF-1α mRNA expression in breast cancers cell lines MCF7 (A and C) and MDA-MB-231 (B and D) transduced with shRNA targeting ΔNp73, or ectopically expressing ΔNp73α (green bars; shΔNp73, blue bars; ΔNp73α) in both normoxia and hypoxia. Samples were normalized to 28S and compared to control (black bars; shCtrl; pcDNA). Relative expression was calculated using the ΔΔCT method; results shown are the mean fold change ± SEM relative to control. n.s., not significant.

**Supplementary Figure 5**: (A-B) Relative fold changes in VHL mRNA expression in ΔNp73 deficient MCF7 and MDA-MB-231 cells (green bars) in normoxia and hypoxia, samples were normalized to 28S and compared to shCtrl (black bars). Relative expression was calculated using the ΔΔCT method; results shown are the mean fold change ± SEM relative to control. n.s., not significant. (C) ChIP-qPCR for p21 show enrichment increase (% input) in MCF7 cells with ectopic expression of pcDNA-HA-ΔNp73α compared to IgG control and pcDNA (empty vector), ****P ≤ 0.0001.
