# Supplementary figures and images for: ΔNp73 enhances HIF-1α protein stability through repression of the ECV complex
Source: Oncogene. 2018 Apr 9;37(27):3729–39. doi: 10.1038/s41388-018-0195-2 (PMC6033838; doi:10.1038/s41388-018-0195-2)

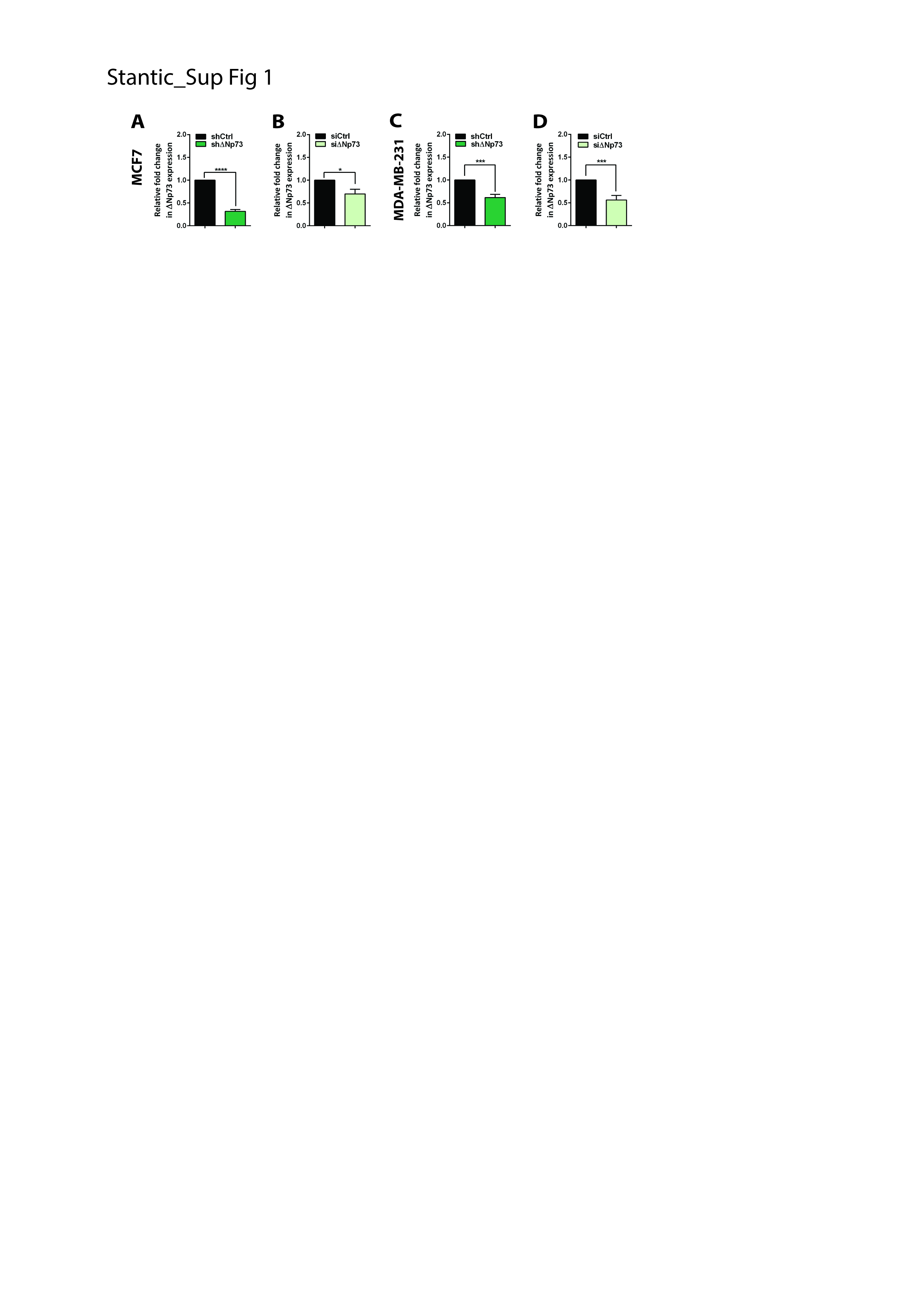

Supplement: Supplementary file 2 — Supplementary Figure 1 [file 41388_2018_195_MOESM2_ESM.tif]

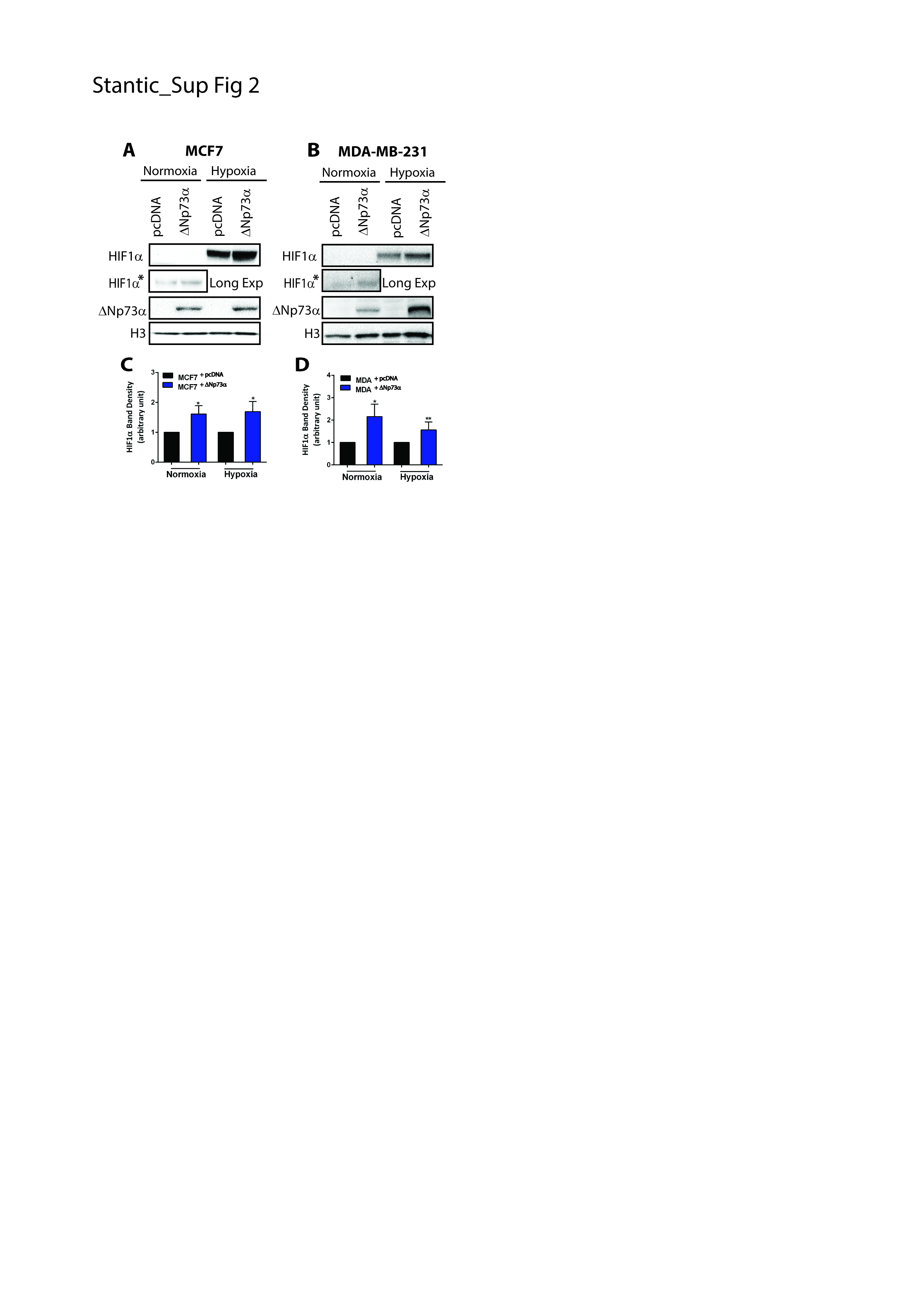

Supplement: Supplementary file 3 — Supplementary Figure 2 [file 41388_2018_195_MOESM3_ESM.tif]

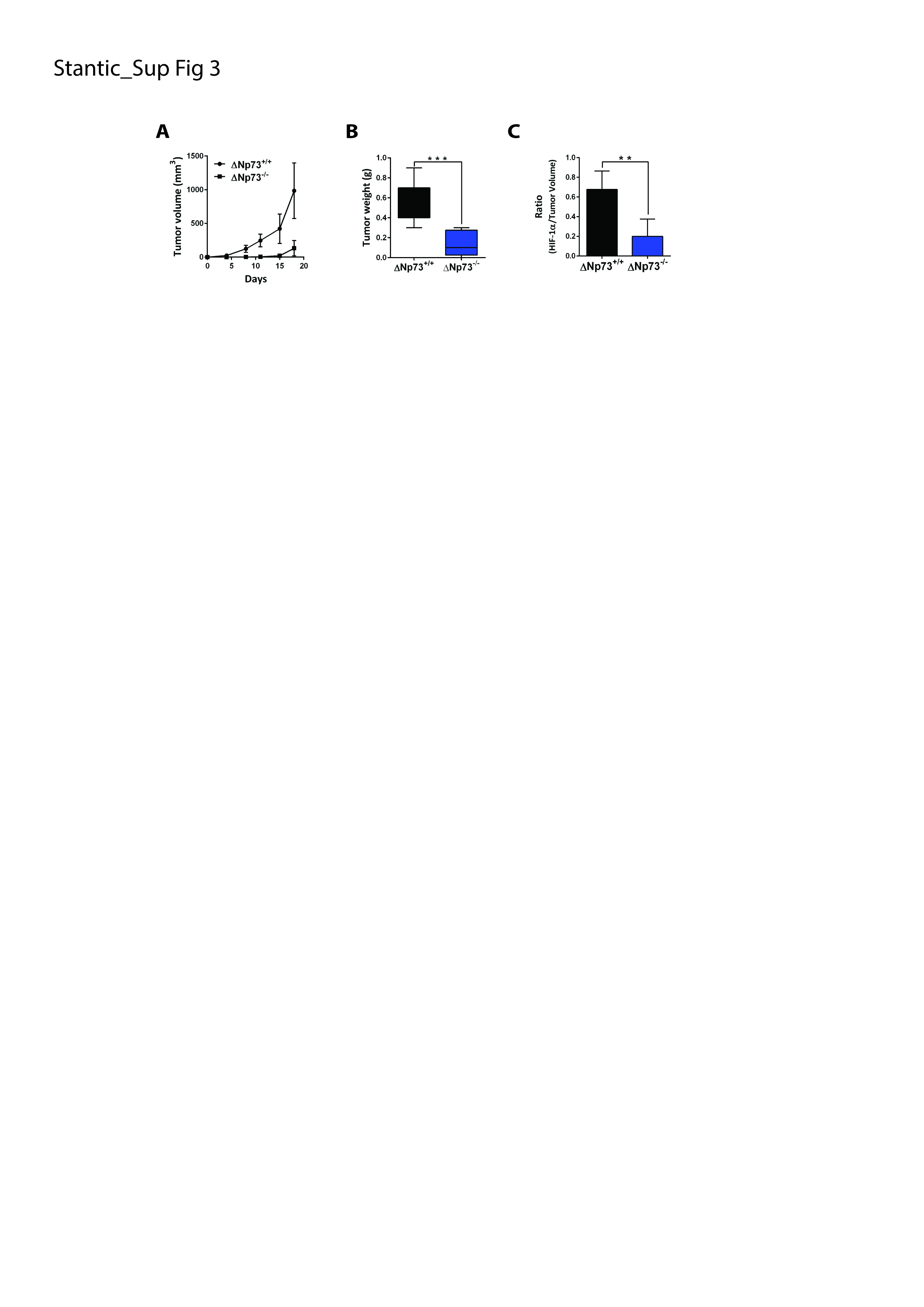

Supplement: Supplementary file 4 — Supplementary Figure 3 [file 41388_2018_195_MOESM4_ESM.tif]

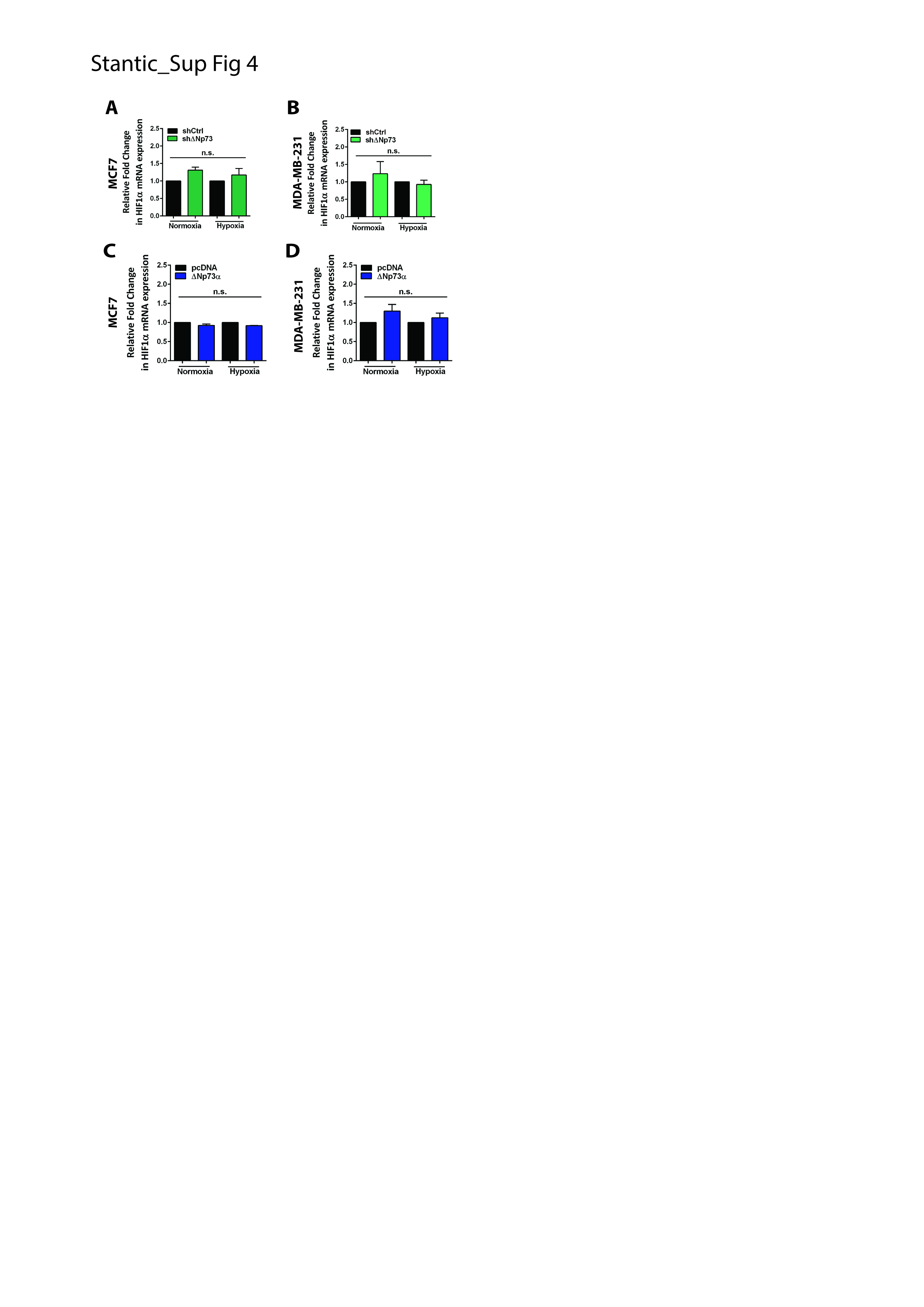

Supplement: Supplementary file 5 — Supplementary Figure 4 [file 41388_2018_195_MOESM5_ESM.tif]

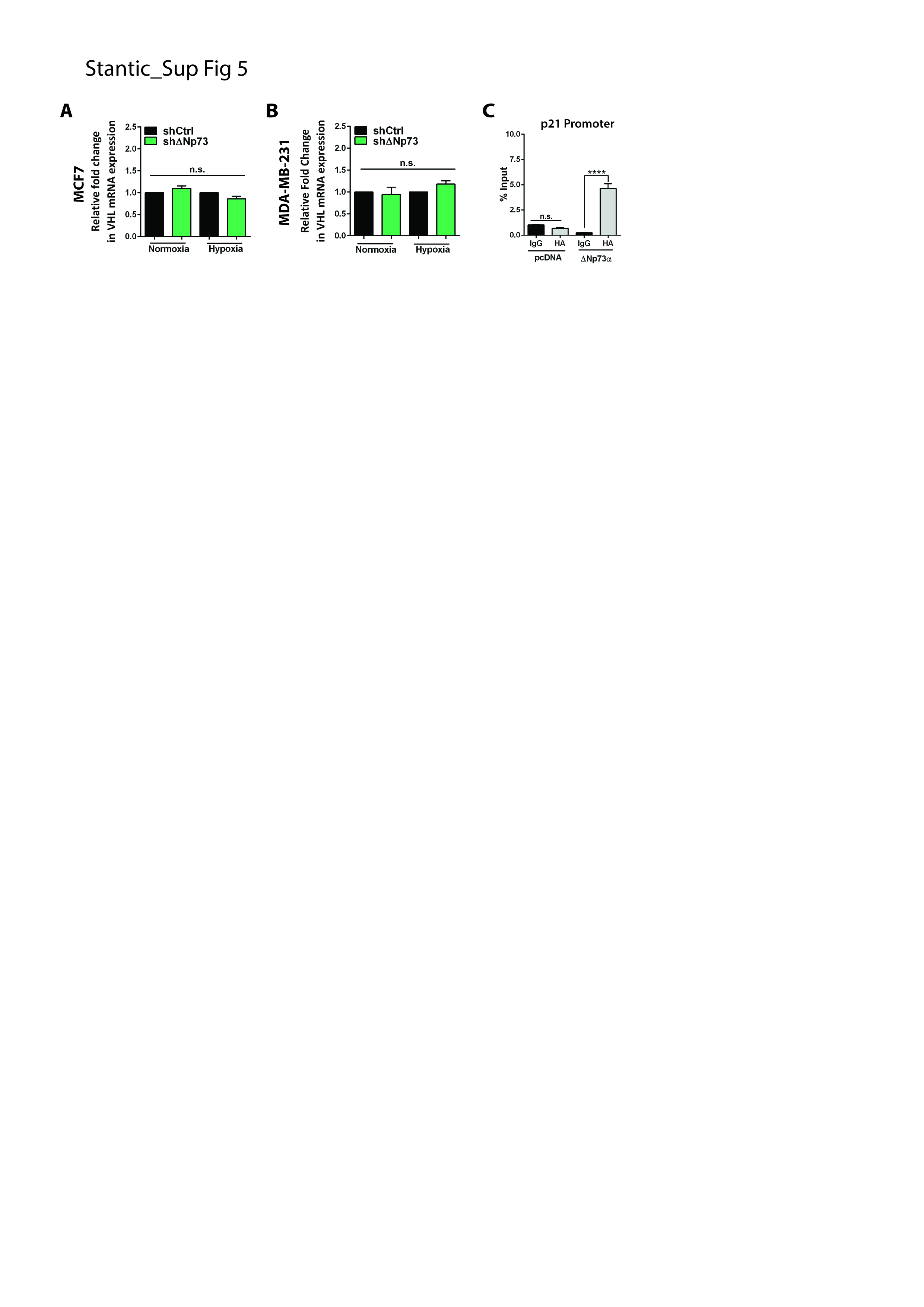

Supplement: Supplementary file 6 — Supplementary Figure 5 [file 41388_2018_195_MOESM6_ESM.tif]
